# Electrochemical Sensing Strategies for Synthetic Orange Dyes

**DOI:** 10.3390/molecules29215026

**Published:** 2024-10-24

**Authors:** Dihua Wu, Jiangwei Zhu, Yuhong Zheng, Li Fu

**Affiliations:** 1 College of Materials and Environmental Engineering, Hangzhou Dianzi University, Hangzhou 310018, China; wudihual@hdu.edu.cn; 2 Co-Innovation Center for Sustainable Forestry in Southern China, Nanjing Forestry University, Nanjing 210037, China; jwzhu@njfu.edu.cn; 3 Institute of Botany, Jiangsu Province and Chinese Academy of Sciences, Nanjing Botanical Garden, Memorial Sun Yat-Sen, Nanjing 210014, China; zhengyuhong@cnbg.net; 4 Jiangsu Key Laboratory for the Research and Utilization of Plant Resources, Nanjing 210014, China

**Keywords:** azo compounds, nanomaterial composites, molecularly imprinted polymers, voltammetry, food safety

## Abstract

This review explores electrochemical sensing strategies for synthetic orange dyes, addressing the growing need for sensitive and selective detection methods in various industries. We examine the fundamental principles underlying the electrochemical detection of these compounds, focusing on their redox behavior and interaction with electrode surfaces. The review covers a range of sensor designs, from unmodified electrodes to advanced nanomaterial-based platforms. Chemically modified electrodes incorporating polymers and molecularly imprinted polymers are discussed for their enhanced selectivity. Particular attention is given to nanomaterial-based sensors, including those utilizing carbon nanotubes, graphene derivatives, and metal nanoparticles, which have demonstrated exceptional sensitivity and wide linear ranges. The potential of biological-based approaches, such as DNA interaction sensors and immunosensors, is also evaluated. Current challenges in the field are addressed, including matrix effects in complex samples and long-term stability issues. Emerging trends are highlighted, including the development of multi-modal sensing platforms and the integration of artificial intelligence for data analysis. The review concludes by discussing the commercial potential of these sensors in food safety, environmental monitoring, and smart packaging applications, emphasizing their importance in ensuring the safe use of synthetic orange dyes across industries.

## 1. Introduction

Synthetic orange dyes have become ubiquitous colorants in various industries, ranging from textiles and food to cosmetics and pharmaceuticals [1]. These vibrant compounds are prized for their stability, cost-effectiveness, and ability to impart rich hues to a wide array of products. However, the widespread use of synthetic orange dyes has raised significant concerns regarding their potential impact on human health and the environment [2]. As a result, there is a growing need for sensitive, selective, and reliable methods to detect and quantify these dyes in diverse matrices. The family of synthetic orange dyes encompasses a variety of chemical structures, with azo compounds being particularly prevalent. These dyes are characterized by their azo linkages (-N=N-) and often contain additional chromophores and auxochromes that contribute to their intense coloration [3]. The structural diversity within this group of dyes presents both challenges and opportunities in terms of detection and analysis [4]. Table 1 shows the list of common orange dyes with their structures.

The importance of detecting synthetic orange dyes stems from several critical factors. Firstly, many of these compounds have been associated with adverse health effects, including allergic reactions, hyperactivity in children, and potential carcinogenicity [5]. Regulatory bodies worldwide have established strict limits on the use of certain synthetic dyes in consumer products, necessitating accurate monitoring methods [6]. Secondly, the illicit use of banned dyes or the adulteration of products with excessive amounts of permitted dyes remains a persistent issue in global trade [7]. This underscores the need for robust analytical techniques capable of identifying and quantifying these compounds in complex matrices. Moreover, the environmental impact of synthetic orange dyes has garnered increasing attention. These compounds can persist in aquatic ecosystems, potentially disrupting the ecological balance and posing risks to aquatic life [7]. The ability to detect trace levels of these dyes in environmental samples is crucial for assessing pollution levels and implementing effective remediation strategies [8]. As such, the development of sensitive and specific detection methods for synthetic orange dyes has become a priority in analytical chemistry and environmental monitoring.

Among the various analytical approaches available, electrochemical sensing strategies have emerged as powerful tools for the detection of synthetic orange dyes [9]. Electrochemical methods offer several advantages, including high sensitivity, rapid response times, potential for miniaturization, and the ability to perform in situ measurements [10]. These techniques exploit the electroactive nature of many synthetic dyes, allowing for their direct detection through redox reactions at electrode surfaces [11]. The fundamental principle underlying the electrochemical sensing of synthetic orange dyes involves the application of an electrical potential to an electrode system, which induces electron transfer between the electrode and the target analyte [12]. This electron transfer process generates a measurable current that is proportional to the concentration of the dye [13]. By carefully controlling the applied potential and optimizing the electrode materials, it is possible to achieve highly selective and sensitive detection of specific dyes even in the presence of interfering substances [14].

Electrochemical sensing techniques encompass a wide range of methodologies, each with its own strengths and applications [15]. Voltametric methods, such as cyclic voltammetry (CV), differential pulse voltammetry (DPV), and square wave voltammetry (SWV), are particularly well-suited for the analysis of synthetic dyes due to their ability to provide both qualitative and quantitative information [16,17]. These techniques can reveal characteristic redox peaks that serve as fingerprints for individual dyes, while also allowing for precise concentration measurements based on peak currents [18]. The development of electrochemical sensors for synthetic orange dyes has been marked by continuous innovation in electrode materials and surface modifications. Unmodified electrodes, such as glassy carbon, gold, and platinum, have demonstrated capability in dye detection but often suffer from limitations in selectivity and sensitivity [19]. To overcome these challenges, researchers have explored a myriad of electrode modification strategies. Chemically modified electrodes, incorporating polymers, self-assembled monolayers, or specific recognition elements, have shown enhanced performance in terms of selectivity and resistance to fouling [20,21]. Nanomaterial-based electrochemical sensors represent a frontier in the field, leveraging the unique properties of materials at the nanoscale to achieve unprecedented sensitivity and selectivity [22,23]. Carbon nanotubes, graphene, and metal nanoparticles have been extensively investigated as electrode modifiers for dye sensing [24]. These nanomaterials not only increase the effective surface area of the electrode but can also catalyze electron transfer reactions, leading to improved analytical performance [25].

This review aims to provide a comprehensive overview of the electrochemical sensing strategies for synthetic orange dyes. We will explore the fundamental principles underlying these techniques, examine the various types of electrochemical sensors developed for dye detection, and highlight recent advances that have pushed the boundaries of sensitivity and selectivity. Additionally, we will discuss the challenges facing the field and outline future perspectives for research and development. By synthesizing the current state of knowledge and identifying emerging trends, this review seeks to guide future efforts in the development of innovative electrochemical sensing platforms for synthetic orange dyes, ultimately contributing to improved food safety, environmental protection, and public health.

## 2. Properties and Classification of Synthetic Orange Dyes

### 2.1. Chemical Structures and Properties

Synthetic orange dyes represent a diverse group of compounds characterized by their ability to impart vibrant orange hues to various substrates. These dyes are primarily organic molecules with complex structures, often featuring conjugated systems that contribute to their color-producing properties [26]. The most common structural feature among synthetic orange dyes is the presence of azo groups (-N=N-), which serve as chromophores responsible for their intense coloration [27]. The chemical structures of synthetic orange dyes typically consist of aromatic rings connected by azo linkages, with additional functional groups that influence their solubility, stability, and reactivity [28]. For instance, food yellow 3, a widely used orange dye in the food industry, contains two sulfonate groups that enhance its water solubility [29]. This dye’s structure comprises two benzene rings connected by an azo bond, with one ring bearing a hydroxyl group and the other containing sulfonate groups [30]. Another prominent synthetic orange dye, Orange II, exhibits a similar azo-based structure but with different substituents. It features a naphthyl group connected to a benzene ring via an azo linkage, with a sulfonate group on the naphthyl moiety conferring water solubility [31]. The presence of a hydroxyl group adjacent to the azo bond in Orange II contributes to its characteristic orange color and influences its chemical reactivity [32].

The molecular structures of these dyes directly impact their physical and chemical properties. Most synthetic orange dyes are solid powders at room temperature, with high melting points due to their aromatic nature and intermolecular interactions. Their solubility varies depending on the presence of polar groups, with many food-grade dyes being highly water-soluble to facilitate their incorporation into aqueous systems [33,34]. The stability of synthetic orange dyes is a crucial property that influences their applications and environmental persistence [35]. Many of these dyes exhibit remarkable stability to light, heat, and pH changes, which makes them desirable for use in various products but also problematic from an environmental perspective [36]. The azo bonds in these dyes are generally resistant to oxidative and reductive conditions, contributing to their longevity in the environment.

**Figure 1:**
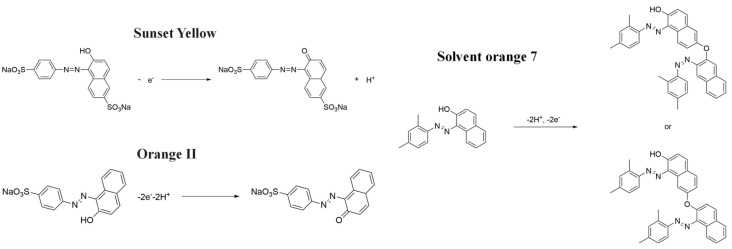
Typical electrochemical reaction mechanism for orange dyes.

The electrochemical properties of synthetic orange dyes are particularly relevant to their detection and analysis. Many of these compounds undergo reversible or quasi-reversible redox reactions, which form the basis for their electrochemical sensing [37,38]. The specific redox potentials and electron transfer kinetics vary among different dyes, providing a means for their selective detection and identification [39]. For example, Figure 1 shows some typical electrochemical reaction mechanisms for orange dyes.

### 2.2. Classification of Synthetic Orange Dyes

**Figure 2:**
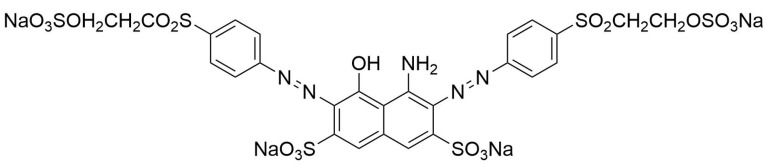
Chemical structure of an azo dye.

Synthetic orange dyes can be classified based on various criteria, including their chemical structure, application method, and regulatory status. One of the most fundamental classifications is based on the chromophore present in the dye molecules [40]. Azo dyes constitute the largest class of synthetic orange dyes. These compounds contain one or more azo groups and can be further subdivided into monoazo, diazo, and polyazo dyes depending on the number of azo linkages present. Generally, the chemical structure of an azo dye is composed of a backbone along with auxochrome groups, chromophoric groups, and solubilizing groups [41,42], as illustrated in Figure 2. The color of azo dyes is primarily determined by the azo bonds in conjunction with their associated chromophores and auxochromes. Examples of azo-based orange dyes include food yellow 3, Orange II, and solvent orange 7 [43]. Azo dyes are prized for their bright colors, ease of synthesis, and versatility in applications.

Anthraquinone dyes represent another important class, although they are less common in orange hues. These dyes are based on the anthraquinone structure and are known for their excellent lightfastness [44,45]. While more prevalent in red and blue colorants, some anthraquinone derivatives can produce orange shades [46]. For example, Quinizarin is an anthraquinone derivative that, while primarily known for its red hues, can be modified to produce various shades, including orange. Its chemical structure allows for substitutions that can shift its color properties [47,48]. Disperse Orange 11 is another example of an anthraquinone dye used to achieve orange shades. It is a synthetic dye primarily used in the textile industry for dyeing polyester and other synthetic fibers [49]. The dye’s molecular structure includes an anthraquinone core with specific substituents that enable it to display vibrant orange colors while maintaining good lightfastness and washfastness, which are critical for textile applications [50]. Vat Orange 1 is also derived from the anthraquinone structure. It is used in vat dyeing processes, where the dye is insoluble in water and must be reduced to a soluble form before application [51]. Once applied, it is oxidized to its original insoluble form within the fiber, ensuring excellent colorfastness. Vat Orange 1 is valued for its bright orange hue and durability, making it suitable for use in fabrics that require high resistance to fading and washing [52]. These examples illustrate the versatility of anthraquinone dyes in producing a range of colors, including orange, through structural modifications and specific application techniques.

Triphenylmethane dyes, while more commonly associated with blue and violet hues, can also yield orange colors through specific structural modifications [53]. These dyes are characterized by their three phenyl rings connected to a central carbon atom.

Another classification system for synthetic orange dyes is based on their solubility and application method. Water-soluble dyes, such as acid dyes, direct dyes, and reactive dyes, are commonly used in the textile industry for dyeing natural fibers [54,55]. Disperse dyes, which have limited water solubility, are utilized for synthetic fibers. Oil-soluble dyes find applications in plastics, oils, and certain cosmetic formulations [56].

Regulatory classifications also play a significant role, particularly in the food industry [57]. In the European Union, food dyes are assigned E numbers, with food yellow 3 designated as E110 [58]. The United States Food and Drug Administration (FDA) uses a different system, referring to certified color additives by FD&C numbers, such as FD&C Yellow No. 6 for food yellow 3 [59].

### 2.3. Applications and Prevalence in Industry

Synthetic orange dyes find widespread applications across various industries due to their vibrant color, stability, and cost-effectiveness [60]. The food industry is one of the largest consumers of synthetic orange dyes, utilizing them to enhance the appearance of a wide range of products. Food yellow 3, for instance, is commonly used in beverages, confectioneries, cereals, and processed foods to impart an attractive orange color [61]. The prevalence of these dyes in the food sector is significant, with global consumption of food yellow 3 alone estimated to be in the thousands of tons annually.

The textile industry represents another major application area for synthetic orange dyes. These colorants are used to dye natural and synthetic fibers, producing a wide range of orange shades in clothing, upholstery, and other textile products [62]. Acid orange dyes are particularly important in this sector, offering good fastness properties and bright hues on wool, silk, and polyamide fibers [63,64]. The global textile dye market, including orange dyes, is a multi-billion dollar industry, reflecting the widespread use of these colorants.

In the cosmetics and personal care industry, synthetic orange dyes play a crucial role in creating visually appealing products [65]. They are used in a variety of formulations, including lipsticks, nail polishes, hair dyes, and soaps. The stability of these dyes to light and pH changes makes them particularly suitable for cosmetic applications where long-lasting color is desired [66].

The plastics and polymer industry utilizes synthetic orange dyes to color a wide range of products, from packaging materials to consumer goods. These dyes are often incorporated directly into the polymer matrix during manufacturing, providing uniform and stable coloration [67,68]. The demand for colorants in this sector continues to grow with the expanding global plastics market. Synthetic orange dyes also find applications in the paper and printing industries, where they are used to produce colored papers, inks, and toners [69]. The ability of these dyes to provide bright and consistent colors is crucial for high-quality printing applications. In the pharmaceutical industry, synthetic orange dyes are used for coating tablets and capsules, serving both aesthetic and functional purposes. They help in product identification and can also play a role in protecting light-sensitive formulations [70].

## 3. Electrochemical Sensing Principles

### 3.1. Fundamentals of Electrochemical Detection

Electrochemical detection is a powerful analytical approach that exploits the intrinsic redox properties of target analytes to achieve sensitive and selective measurements [71]. At its core, electrochemical sensing relies on the transfer of electrons between an electrode surface and electroactive species in solution [72]. This electron transfer process generates an electrical signal that can be measured and correlated with the concentration of the analyte. The fundamental components of an electrochemical sensing system typically include a working electrode, where the primary electrochemical reactions occur; a reference electrode, which maintains a stable potential; and often a counter electrode to complete the electrical circuit. The working electrode serves as the transducer, converting chemical information into an electrical signal. The choice of electrode material is crucial, as it influences the sensitivity, selectivity, and overall performance of the sensor [73,74].

When an appropriate potential is applied to the working electrode, it can either oxidize or reduce the electroactive species present in the sample. The electrochemical behavior of analytes is governed by several key factors, including mass transport, electron transfer kinetics, and surface adsorption phenomena [75]. Mass transport, which involves the movement of analyte molecules to and from the electrode surface, can occur through diffusion, convection, or migration [76]. In most electrochemical sensing applications for dyes, diffusion is the primary mode of mass transport [77]. Electron transfer kinetics play a crucial role in determining the shape and magnitude of the electrochemical response. Fast electron transfer processes result in reversible electrochemical behavior, characterized by well-defined peaks in voltametric measurements. Slower kinetics can lead to quasi-reversible or irreversible behavior, which may require different interpretation approaches [78].

The surface adsorption of analytes on the electrode can significantly impact the electrochemical response. For many synthetic orange dyes, adsorption processes can enhance the sensitivity of detection by concentrating the analyte at the electrode surface. However, strong adsorption can also lead to electrode fouling, necessitating careful electrode preparation and cleaning procedures [79,80]. The selectivity of electrochemical sensors is often achieved through the careful selection of operating potentials, electrode materials, and surface modifications. For complex samples containing multiple electroactive species, techniques such as differential pulse voltammetry or square wave voltammetry can enhance selectivity by minimizing background currents and resolving overlapping signals [81].

### 3.2. Electrochemical Behavior of Synthetic Orange Dyes

The electrochemical behavior of synthetic orange dyes is intrinsically linked to their molecular structure, with the azo group (-N=N-) serving as the primary electroactive center in most cases [82]. The reduction of azo bonds typically occurs in a stepwise manner, often involving the transfer of four electrons per azo group to form the corresponding amine products [83]. This process can be influenced by the pH, with acidic conditions typically favoring reduction.

For monoazo dyes, such as Orange II, the electrochemical reduction generally proceeds through a two-step mechanism. The first step involves a two-electron, two-proton transfer to form a hydrazo intermediate. This is followed by a second two-electron, two-proton transfer to cleave the N-N bond, resulting in the formation of aromatic amines [84]. The exact potentials at which these reductions occur depend on the specific molecular structure, substituents, and the electrode material.

Diazo and polyazo dyes exhibit more complex electrochemical behavior due to the presence of multiple azo groups [85]. These compounds often show multiple reduction peaks corresponding to the sequential reduction of each azo bond. The relative positions and intensities of these peaks can provide valuable information for dye identification and quantification in mixtures.

The presence of other electroactive groups in the dye molecule, such as hydroxyl or amino substituents, can also contribute to the overall electrochemical response. These groups may undergo oxidation or reduction processes at potentials distinct from the azo group reduction, potentially offering additional analytical signals or complicating the interpretation of voltametric data [86,87].

Adsorption phenomena play a significant role in the electrochemical behavior of many synthetic orange dyes [88]. Strong adsorption onto electrode surfaces can lead to enhanced sensitivity but may also result in electrode fouling or non-linear calibration curves at higher concentrations [89]. The extent of adsorption is influenced by factors such as the hydrophobicity of the dye, the nature of the electrode material, and the solution conditions [90]. The pH of the supporting electrolyte has a profound effect on the electrochemical behavior of synthetic orange dyes. In general, the reduction of azo bonds is facilitated in acidic media due to the involvement of protons in the reduction mechanism. The peak potentials of the reduction processes typically shift to more positive values with decreasing pH [91]. However, extremely low pH conditions can lead to the chemical instability of some dyes, necessitating careful optimization of the electrolyte composition.

## 4. Electrochemical Sensors for Synthetic Orange Dyes

### 4.1. Unmodified Electrode Sensors

Unmodified electrode sensors represent the most straightforward approach to the electrochemical detection of synthetic orange dyes [92]. These sensors typically employ conventional electrode materials such as glassy carbon, carbon paste, gold, or platinum without any additional surface modifications. The simplicity of these systems offers advantages in terms of reproducibility, ease of preparation, and cost-effectiveness [93].

The static mercury drop electrode (SMDE) has been used in the early stage for studying the electrochemical behavior of certain orange dyes. For example, square wave adsorptive stripping voltammetry was employed to determine trace amounts of the dye food yellow 3 [94]. Researchers investigated its voltametric behavior at various pH levels, optimizing conditions to achieve sensitive and accurate measurements. The study utilized a static mercury drop electrode, which provided a significant advantage in detecting food yellow 3 due to its ability to enhance sensitivity through adsorptive stripping techniques. The method demonstrated a linear calibration range from 5 to 90 µg/L with a relative standard deviation of 2.2% for a 30 µg/L solution, highlighting its precision. The detection limit was established at 5 µg/L after a 15 s accumulation at −0.40 V. This approach proved effective in analyzing food yellow 3 in commercial drinks, yielding results comparable to those obtained via HPLC with spectrophotometric detection. The SMDE’s role was crucial in achieving high sensitivity and selectivity, enabling rapid analysis with minimal sample preparation. Similar work has also been conducted for acridine orange [95,96].

**Figure 3:**
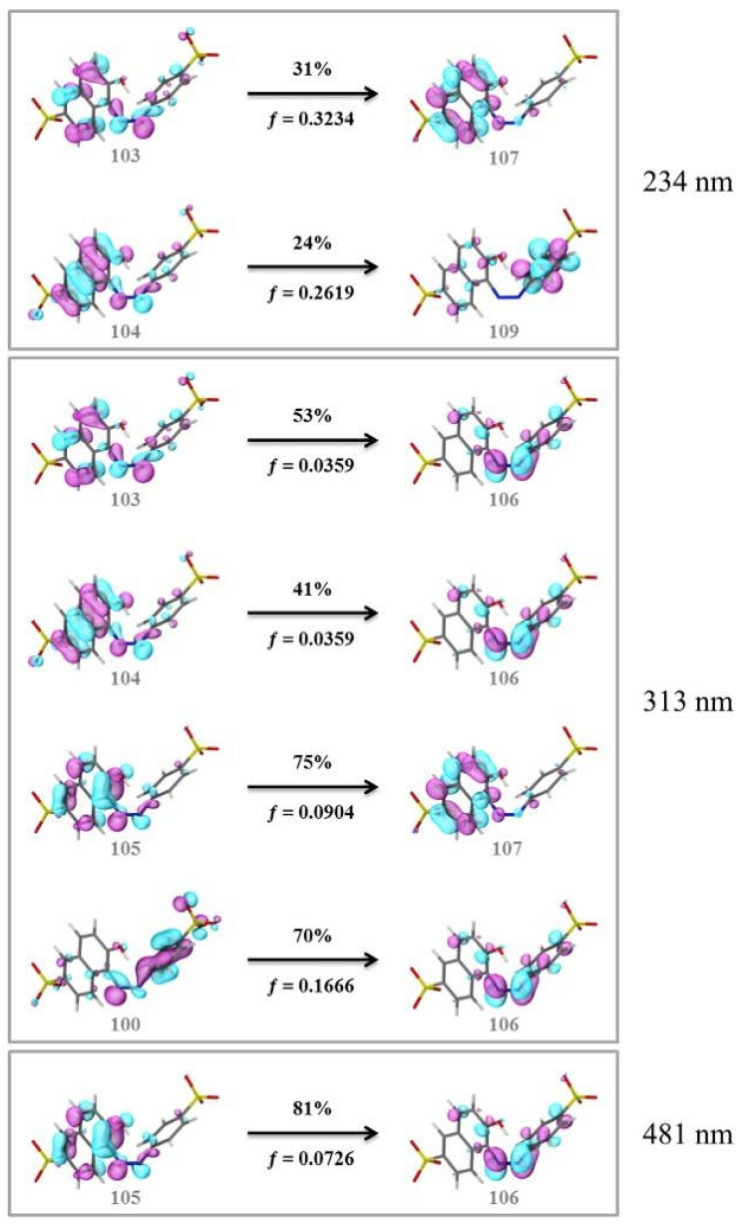
Active molecular orbitals in the electronic transition of food yellow 3 [97].

Glassy carbon electrodes (GCEs) have been widely utilized for the detection of various synthetic orange dyes due to their broad potential window, low background current, and chemical inertness. When applied to the sensing of azo dyes, GCEs often exhibit well-defined reduction peaks corresponding to the stepwise reduction of azo bonds. Sierra-Rosales et al. [97] investigated the electrooxidation pathways of food yellow 3 using GCEs in a phosphate buffer solution at pH 7.0. Researchers employed a combination of electrochemical methods, UV–Vis spectroscopy, and density functional theory calculations to elucidate the oxidation mechanisms. The oxidation potential for food yellow 3 was experimentally found to be 0.64 V versus Ag/AgCl, which was lower than the theoretical prediction of 0.88 V for a single electron transfer, indicating a more complex mechanism involving proton release that reduced the potential to 0.43 V. The study analyzed the active molecular orbitals involved in the electronic transitions of food yellow 3 (Figure 3). The transition around 490 nm was primarily an n-π* transition, dominated by the movement of electrons from a non-bonding orbital on the azo group (HOMO) to an anti-bonding π* orbital (LUMO) also centered on the azo moiety. This transition indicated that the azo group played a crucial role in the electronic structure of food yellow 3. Additionally, transitions around 315 nm involved π-π* transitions, with electron movement from the aromatic rings to the azo group’s π* orbital, further highlighting the azo group’s significance. The study proposed a new reaction pathway for food yellow 3, highlighting the azo group’s oxidation, which led to the loss of color and structural modifications in the azo moiety. GCEs have also been used to study the electrochemical behavior of acid orange 7 [98]. The researchers gained new insights into the mechanistic details, pH dependence, and intermediate structures of both the oxidation and reduction processes of acid orange 7. Surprisingly, they found that the same redox couple (1-iminonaphthalen-2(1H)-one/1-aminonaphthalen-2-ol) formed from both the oxidation and reduction of acid orange 7. The study also focused on the electrochemical synthesis of three new derivatives of 1-amino-4-(phenylsulfonyl)naphthalen-2-ol under constant current electrolysis. The synthesis was carried out in an undivided cell with carbon rod electrodes, achieving yields of 60–65% for the new compounds. The researchers observed that the electrogenerated 1-iminonaphthalen-2(1H)-one participated in a Michael addition reaction with arylsulfinic acids to form the desired products.

Lu et al. [99] developed a sensitive electrochemical sensor for detecting food yellow 3 using an electrochemically activated GCE (AGCE). The AGCE was fabricated through CV, which generated oxygen-containing functional groups on the electrode surface, improving its conductivity and electron transfer rate. This modification led to a significant increase in the oxidation peak current, nearly tenfold compared to a non-activated GCE. The sensor demonstrated a linear detection range from 0.005 to 1.0 µM and achieved a low detection limit of 0.00167 µM. The AGCE’s performance was superior to other methods, showing excellent repeatability with an RSD of 4.51% and reproducibility with an RSD of 2.45%. It also exhibited strong anti-interference capabilities and was successfully applied to detect food yellow 3 in beverage samples, achieving recovery rates between 96.19% and 103.47%. The performance of GCEs can be influenced by surface pretreatment procedures, such as mechanical polishing or electrochemical activation, which can enhance sensitivity by increasing the active surface area or introducing surface functional groups. Romanini et al. [100] developed a simple and rapid electroanalytical method for determining solvent orange 7 dye. They optimized a square-wave voltametric technique using a glassy carbon electrode in a mixture of Britton–Robinson buffer and N,N-dimethylformamide. They found that solvent orange 7 exhibited a well-defined oxidation peak at +0.70 V vs. Ag/AgCl. The method demonstrated a linear relationship between the peak current and solvent orange 7 concentration from 4 to 18 μM. The LOD was calculated to be 0.9 μM, indicating good sensitivity. When applied to commercial fuel ethanol samples spiked with 5.0 mg/L and 10.0 mg/L of solvent orange 7, the method achieved recovery rates of 97.20% and 98.40%, respectively, with relative standard deviations of 5.60% and 3.30%.

### 4.2. Chemically Modified Electrode Sensors

Chemically modified electrodes (CMEs) represent a significant advancement in the field of electrochemical sensing for synthetic orange dyes [101]. These sensors incorporate various chemical modifiers on the electrode surface to enhance performance characteristics such as sensitivity, selectivity, and stability. The modifiers can range from simple organic molecules to complex polymers, each tailored to interact specifically with target dye molecules. For example, Zougar et al. [102] developed and characterized an impedimetric sensor for detecting acid orange 10 in polluted industrial wastewater. The researchers used a tridodecylamine (TDA) as the ionophore, deposited on a gold electrode. The sensor exhibited a wide linear response range from 0.1 pM to 1 mM for acid orange 10 in aqueous medium, with a correlation coefficient of 0.9862 and an exceptional LOD of 1 pM. Tridodecylamine was chosen for its excellent chemical stability and efficiency in extracting azo dyes. The sensor demonstrated good selectivity against methyl blue interference at concentrations above 10 nM.

Polymer-modified electrodes have gained considerable attention for dye sensing applications [103]. These polymers not only increase the effective surface area of the electrode but can also participate in electrocatalytic processes, enhancing the electron transfer kinetics. For instance, an electrochemical sensor was developed using a poly(aminosulfonic acid)-modified GCE (PASA/GCE) to determine the presence solvent orange 7 in food samples [104]. The purpose was to create a sensitive, reliable, and convenient method for detecting this carcinogenic dye. The PASA/GCE was fabricated through electropolymerization, and its electrochemical behavior was analyzed using CV. The results indicated that the modified electrode exhibited significant electrocatalytic activity towards the redox reaction of solvent orange 7. Under optimal conditions, the oxidation peak current showed a linear relationship with solvent orange 7 concentration in the ranges of 4.0 × 10^−^⁸ to 1.0 × 10^−^⁶ M and 1.0 × 10^−^⁶ to 1.2 × 10^−^⁵ M, with an LOD reaching 4.0 × 10^−^⁹ M. The poly(riboflavin)-modified carbon paste sensor (PRFMCPS) can be used for detecting methyl orange [105]. The purpose of using poly(riboflavin) was to enhance the electrocatalytic activity and sensitivity of the electrode for methyl orange detection. The PRFMCPS exhibited a higher peak current and lower charge transfer resistance compared to the bare carbon paste sensor, indicating improved electrocatalytic performance. The optimal pH for methyl orange detection was determined to be 6.5. The sensor demonstrated a linear range of 0.6–8.0 μM with an LOD of 0.699 μM and an LOQ of 0.023 μM. The PRFMCPS showed good stability, with 94.96% retention of its initial response after 50 consecutive CV cycles.

**Figure 4:**
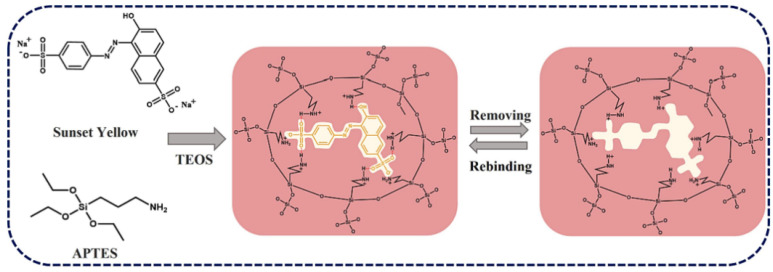
Scheme for the preparation of RMIEC with APTES as functional monomers and food yellow 3 as templates [106].

Another approach in CME design involves the use of molecularly imprinted polymers (MIPs). These synthetic materials are created with specific recognition sites that match the size, shape, and functionality of target dye molecules. MIP-modified electrodes have demonstrated remarkable selectivity in the detection of specific orange dyes, even in the presence of structurally similar compounds. The high specificity of MIPs makes them particularly valuable for applications in complex food and environmental samples where multiple interfering species may be present. Wu et al. [106] developed a ratiometric MIP electrochemical sensor (RMIEC) for detecting food yellow 3. The sensor utilized molecularly imprinted polymers (MIPs) to achieve high specificity and sensitivity. AuNPs were incorporated to enhance electron transfer and serve as a reference signal. The RMIEC demonstrated a wide linear detection range from 10 nM to 100 μM with a low detection limit of 1.60 nM. This dual-signal output mode improved detection stability and sensitivity by providing a built-in calibration. The study highlighted the sensor’s applicability in real food samples, achieving recovery rates between 94.0% and 97.0% with relative errors ranging from 5.4% to 8.3%. Compared to traditional electrochemical sensors, the RMIEC offered a superior performance due to the integration of MIPs, which provided specific recognition of food yellow 3 and allowed for convenient enrichment and separation (Figure 4). Arvand et al. [107] developed an electrochemical sensor using MIPs on functionalized multi-walled carbon nanotubes (f-MWCNTs) for the selective recognition of food yellow 3 in food samples. The sensor was fabricated by electropolymerizing MIPs in the presence of food yellow 3 onto a GCE. It demonstrated excellent recognition capabilities compared to other structurally similar molecules. Under optimal conditions, the sensor exhibited a linear current response to food yellow 3 concentrations ranging from 0.05 to 100 µM, with an LOD of 5.0 nM. This performance was attributed to the increased surface area, mechanical strength, and electrical conductivity provided by the MIP and f-MWCNTs. MIP has also been used for basic orange 1 detection [108]. Magnetic graphene oxide/β-cyclodextrin/gold nanoparticle (MGO/β-CD@AuNPs) composites have been used to modify GCEs. MIPs were then electropolymerized on the modified electrode surface using basic orange 1 as the template molecule. The resulting sensor exhibited an excellent analytical performance, with a wide linear detection range of 50 nM to 50 μM and a low detection limit of 17 nM. The sensor demonstrated high selectivity, good sensitivity, and acceptable reproducibility when applied to basic orange 1 detection in spiked water samples. The MGO/β-CD@AuNPs composite enhanced the sensing surface area and electronic transmission rate, while the MIPs provided specific recognition sites for chrysoidine.

### 4.3. Nanomaterial-Based Electrochemical Sensors

The advent of nanotechnology has revolutionized the field of electrochemical sensing, offering new possibilities for the detection of synthetic orange dyes [109]. Nanomaterial-based sensors leverage the unique properties of materials at the nanoscale, including high surface area-to-volume ratios, enhanced electrocatalytic activity, and novel electron transfer properties [110]. Carbon nanomaterials have emerged as particularly promising candidates for dye sensing applications. Carbon nanotubes (CNTs), both single-walled and multi-walled varieties, have been extensively studied as electrode modifiers. When incorporated into sensor designs, CNTs can significantly enhance the electroactive surface area and promote rapid electron transfer. For the detection of orange azo dyes, CNT-modified electrodes have demonstrated improved sensitivity and lower detection limits compared to their unmodified counterparts. The π-π interactions between the CNT surface and the aromatic structures of dye molecules can also contribute to preconcentration effects, further enhancing sensitivity. For example, Irfan et al. [111] described an electrochemical sensor for detecting Orange II dye using a GCE modified with amino-functionalized multi-walled carbon nanotubes (NH_2_-fMWCNTs) and zinc oxide nanoparticles. The researchers aimed to create a cost-effective and rapid method for analyzing Orange II in water samples. The NH_2_-fMWCNTs played a crucial role by increasing the electrode’s surface area and facilitating the preconcentration of the negatively charged dye molecules in acidic medium. Under optimized conditions, including a phosphate buffer solution at pH 6.0, 0.2 V deposition potential, and 20 s deposition time, the sensor exhibited an excellent performance. It achieved a remarkably low detection limit of 0.57 nM and a quantification limit of 1.92 nM. The sensor demonstrated a wide linear range from 0.03 to 0.09 μM with a correlation coefficient of 0.998. The sensor successfully detected Orange II in real samples such as industrial wastewater, fruit juice, and ketchup sauce with recovery rates ranging from 96.00% to 98.33%, indicating its practical applicability in complex matrices.

**Figure 5:**
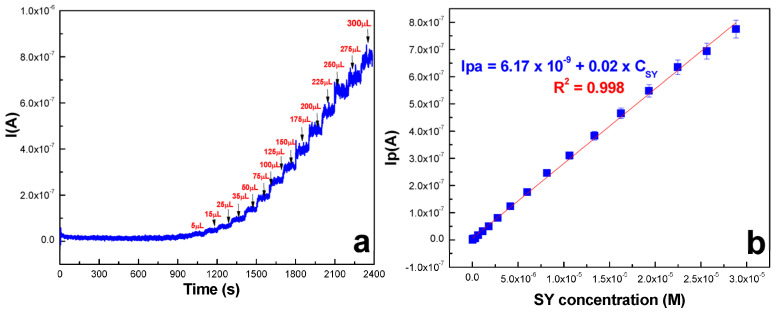
(**a**) Amperometric curves recorded with GR-exf/GCE after the addition of food yellow 3 from 0.028 to 30 µM; (**b**) the corresponding calibration plot [112].

Graphene and its derivatives represent another class of carbon nanomaterials that have shown great potential in electrochemical dye sensing [109]. The two-dimensional structure of graphene provides an exceptionally high surface area for dye adsorption, while its excellent electrical conductivity facilitates rapid electron transfer. Reduced graphene oxide (rGO), in particular, has been successfully employed in sensors for various synthetic orange dyes, often exhibiting wider linear ranges and lower detection limits than conventional electrode materials. Măgeruşan et al. [112] investigated the use of a GCE modified with few-layer graphenes for detecting the food yellow 3 food colorant. They produced graphene through electrochemical exfoliation of graphite rods (GR-exf) and characterized it using various techniques. The modified electrode exhibited enhanced catalytic activity towards food yellow 3 oxidation compared to bare electrodes. In pH 6 phosphate buffer solution, the sensor showed a quasi-reversible process over a wide food yellow 3 concentration range of 1–100 μM. Using cyclic voltammetry and square wave voltammetry, the limit of detection was 0.303 μM, while amperometry yielded a lower detection limit of 0.0085 μM (Figure 5). The sensor demonstrated a good stability, retaining 83.56% of its original signal after 50 successive scans and 83.07% after 120 days of storage. Yun et al. [113] proposed an electrochemical sensor for Orange II detection using electrochemically reduced GO grafted with 5-amino-1,3,4-thiadiazole-2-thiol-Pt nanoparticles (ERGO-ATDT-Pt) on a GCE. The ERGO-ATDT-Pt modified electrode exhibited superior electrocatalytic activity towards Orange II oxidation compared to unmodified electrodes. CV and chronoamperometry measurements revealed a wide linear range of 10 nM to 600 nM and an LOD of 0.34 nM for Orange II sensing. The graphene-based composite enhanced electron transfer and increased the electroactive surface area, leading to improved sensitivity. Real sample analysis in food products showed high recoveries (>97%) with relative standard deviations below 5%.

**Figure 6:**
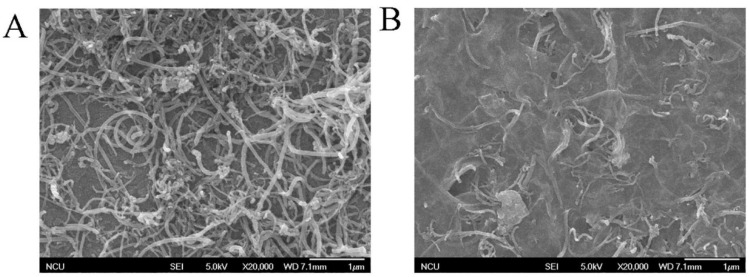
The SEM images of MWCNTs (**A**) and GO/MWCNTs (**B**) films [114].

The combination of graphene and CNT can further enhance the sensing performance. For example, Qiu et al. [114] developed an electrochemical platform for the simultaneous detection of food yellow 3 by combining GO and MWCNTs. They created a stable GO/MWCNTs composite through sonication mixing, which exhibited superior performance compared to individual GO or MWCNTs electrodes (Figure 6). The composite electrode demonstrated a strong enhancement effect and significantly increased the oxidation signals of food yellow 3. Under optimized conditions, the sensor showed excellent analytical performance for the detection of food yellow 3 in the range of 0.09–8.0 μM, with low detection limits of 0.025 μM for food yellow 3. The synergistic effect of GO’s signal amplification properties and MWCNTs’ excellent electronic and antifouling characteristics resulted in improved sensitivity and selectivity.

Metal nanoparticles, including gold, silver, and platinum, have been widely incorporated into electrochemical sensors for synthetic dyes [115]. These nanoparticles can serve multiple functions, acting as electrocatalysts, increasing the effective surface area, and in some cases, providing unique optical properties for combined electrochemical–optical sensing approaches. Gold nanoparticle-modified electrodes, for instance, have shown enhanced sensitivity towards the oxidation of certain azo dyes, with the nanoparticles facilitating electron transfer and potentially catalyzing dye degradation processes. Ahmadi et al. [116] developed an electrochemical sensor for detecting three artificial food dyes—carmoisine (CR), food yellow 3, and tartrazine (TR)—in fruit juices. They created a novel chemosensor by modifying a gold electrode with a green polymeric nanocomposite of beta-cyclodextrin/arginine decorated with gold nanoparticles-capped cysteamine. It exhibited a wide linear detection range of 10^−8^ to 10^−4^ M for all three dyes, with an LOD of quantification around 1 nM. The sensor showed a good performance in real fruit juice samples, with recovery rates of 70–95% for the dyes at a 0.0001 M concentration. Interference studies revealed that some common juice components like potassium and beta-carotene affected the signals, but many potential interferents had a minimal impact. Carbamazepine-functionalized silver nanoparticles (Cbz-AgNPs) have been used for the simultaneous detection of Orange II as well [117]. The modified GCE (Cbz-AgNP/GCE) demonstrated excellent sensing capabilities, with detection limits of 1.2 nM for Orange II. The sensor exhibited a wide linear range from 1.56 nM to 12 μM. Importantly, the Cbz-AgNP/GCE showed high selectivity, maintaining its performance even in the presence of interfering agents at 200-fold-higher concentrations. The use of AgNPs significantly enhanced the sensor’s sensitivity and electron transfer rate, while the carbamazepine coating improved analyte enrichment through host–guest interactions. Tcheumi and Babu [118] developed an electrochemical sensing platform for methyl orange detection using surfactant-intercalated smectite clay-modified electrodes. They prepared organosmectites by intercalating hexadecyltrimethylammonium cations into smectite clay and characterized them using various techniques. They then evaluated an amperometric sensor based on the organosmectite as an electrode modifier for methyl orange sensing using SWV. The modified electrode showed significantly enhanced oxidation peak currents for methyl orange compared to unmodified electrodes. Under optimized conditions, the sensor exhibited a linear response to methyl orange concentrations from 0.1 to 1.6 μM with an LOD of 40 nM.

Composite nanomaterials, combining two or more nanoscale components, have also been explored for dye sensing applications. Nanocomposites such as CNT–metal nanoparticle hybrids or graphene-conducting polymer composites can synergistically enhance sensor performance, leveraging the unique properties of each component. Liu et al. [119] developed an electrochemical sensor using Fe_2_O_3_ nanoparticles, oxygen-functionalized MWCNTs (MWCNTs-COOH), and Triton X-100 to modify a CPE for the simultaneous detection of Orange G and Orange II in industrial wastewater. The modified electrode (Fe_2_O_3_/MWCNTs-COOH/OP/CPE) exhibited enhanced electrocatalytic activity towards both dyes compared to unmodified electrodes. Under optimized conditions, the sensor demonstrated linear detection ranges of 0.1–20.0 μM for Orange G and 0.2–50.0 μM for Orange II, with LOD of 0.05 μM and 0.1 μM, respectively. It also displayed good repeatability with relative standard deviations of 2.3% for Orange G and 1.7% for Orange II. The sensor retained over 99% of its performance after 60 min of continuous operation and maintained 93.2% and 89.7% of its initial response for Orange G and Orange II, respectively, after 15 days of storage. When applied to real industrial wastewater samples, the sensor achieved recoveries ranging from 96.8% to 105.1% for Orange G and 97.4% to 103.8% for Orange II, demonstrating its practical applicability for environmental monitoring. Gan et al. [120] developed an electrochemical method for detecting Orange II dye using a graphene–mesoporous TiO_2_ nanocomposite-modified GCE. Under optimal conditions, the method achieved a wide linear range of 2.85 nM to 0.48 μM and a low detection limit of 0.92 nM for Orange II after 4 min of accumulation. It demonstrated good reproducibility with 6.4% RSD for 10 electrodes. The method was successfully applied to detect Orange II in food samples like chili powder, chili paste, and ketchup, with results comparable to HPLC-MS analysis.

In addition to traditional nanomaterials, emerging materials such as metal–organic frameworks (MOFs) and MXenes have shown great promise in enhancing the performance of electrochemical sensors for synthetic orange dyes. Table 2 summarizes of electrochemical sensors for acid red dyes detection.

**Figure 7:**
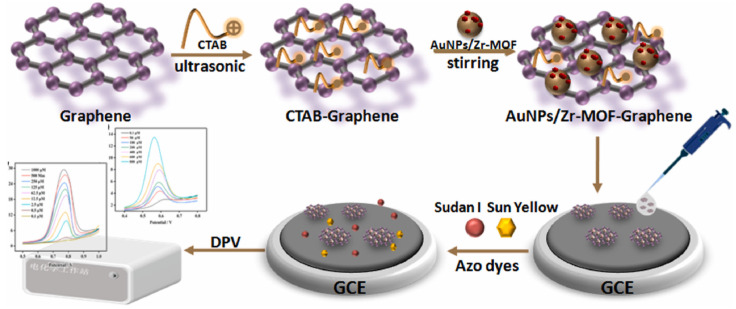
Illustration of AuNPs/Zr-MOF–graphene-modified electrode for the sensing of food yellow 3 [121].

MOFs have garnered significant attention due to their high surface area, tunable pore sizes, and diverse metal centers, which can be tailored to enhance both sensitivity and selectivity in dye detection. For instance, Sun et al. [121] developed a sensitive electrochemical sensor using AuNPs/Zr-MOF–graphene composites for detecting food yellow 3 in food samples (Figure 7). They synthesized the composite material by combining gold nanoparticles, zirconium-based MOF (Zr-MOF), and graphene. The sensor exhibited excellent performance due to the synergistic effects of these components. The Zr-MOF provided a large surface area and porous structure for capturing analytes, while the AuNPs enhanced electron transfer. Under optimal conditions, the sensor achieved wide linear detection ranges of 0.1–1000 μM for food yellow 3, with a low limit of detection of 0.1 μM. The use of Zr-MOF was crucial in improving the sensor’s accumulation efficiency and electrocatalytic activity. The excellent performance was attributed to the high surface area and abundant active sites provided by the MOF structure, as well as the strong π-π interactions between the MOF and the dye molecules.

MXenes, a relatively new class of two-dimensional transition metal carbides and nitrides, have also shown great potential in electrochemical sensing applications. Their excellent electrical conductivity and large surface area make them attractive candidates for dye detection. Wang et al. [122] demonstrated the use of Ti_3_C_2_T_x_ MXene for sensitive detection of Sudan I, achieving a detection limit of 0.27 nM. While this study focused on a different dye, the principles could be readily applied to orange dye detection. The layered structure of MXenes allows for easy intercalation of dye molecules, potentially enhancing sensitivity and selectivity. The integration of these emerging materials with traditional nanomaterials can lead to synergistic effects, further improving sensor performance. For example, Zeng et al. [123] developed a hybrid material combining MXene and TiO_2_/TiN@N-C heterostructure for the detection of food yellow 3. The sensor exhibited a wide linear range from 2.5 to 1250 nM and a detection limit of 1.2 nM.

These emerging materials offer several advantages over traditional nanomaterials in terms of stability, selectivity, and tunability. MOFs, in particular, provide a high degree of structural and chemical tunability, allowing for the design of sensors with tailored properties for specific dye molecules. MXenes offer exceptional stability and conductivity, addressing some of the limitations of graphene-based materials. Moreover, these materials often exhibit improved scalability compared to traditional nanomaterials. For instance, the crystalline nature of MOFs allows for more consistent and reproducible synthesis, potentially facilitating large-scale production of sensors. MXenes can be produced in large quantities through relatively simple exfoliation processes, making them attractive for commercial applications.

While nanomaterials offer significant advantages in electrochemical sensing, their long-term stability and performance can be affected by surface oxidation and aggregation. These issues are particularly relevant for the metal nanoparticles and carbon-based nanomaterials used in orange dye detection. Surface oxidation is a common challenge for metal nanoparticles, such as gold and silver, which are frequently used in dye sensors. Oxidation can lead to decreased conductivity and altered surface chemistry, potentially reducing sensor sensitivity and selectivity over time. The aggregation of nanoparticles is another significant issue, especially for colloidal systems. As particles aggregate, the effective surface area diminishes, leading to reduced sensitivity.

To mitigate these challenges, several strategies have been developed:

- Surface functionalization: Coating nanoparticles with organic ligands or polymers can prevent oxidation and aggregation.
- Core-shell structures: Encapsulating metal nanoparticles in a thin carbon or silica shell can prevent oxidation while maintaining electrochemical activity.
- Stabilizing agents: Incorporating stabilizing agents in the sensor matrix can prevent nanoparticle aggregation.
- Controlled nanostructure engineering: Creating interconnected nanostructures can enhance stability.
- In situ regeneration: Implementing electrochemical cleaning protocols can help to regenerate the sensor surface.

**Table 2:** Summary of electrochemical sensors for acid red dyes detection.

Dye	Sensor	LDR	LOD	Real Sample	Ref.
Food yellow 3	AGCE	0.005 to 1.0 µM	0.00167 µM	Beverage samples	[99]
	SMDE	5 to 90 µg/L	5 µg/L	Refreshing drink	[94]
	MWCNTs@MIP-PDA/GCE	0.0022 to 4.64 μM	0.0014 μM	Jelly, fruit drink, chocolate, instant juice powder, ice cream, candy	[124]
	MIP/f–MWCNTs/ GCE	0.05 to 100 μM	0.005 μM	Candy, candy-coated chocolate, orange flavored jelly powder, peach juice powder, beverage	[107]
	RMIEC	10 nM to 100 μM	1.60 nM	Mirinda orange, Fanta orange	[106]
	P(*β*-CD/Arg)/CysA-AuNPs/AuE	10 nM to 0.1 mM	1 nM	Pomegranate juice, pomegranate nectar, orange nectar, orange drink juice	[116]
	GR-exf/GCE	0.028 to 30 μM	8.5 nM	Pharmaceutical tablets (Triferment), lollipop candy, two different commercial orange juice samples	[112]
	SiO_2_@PDA NPsMIP/CPE	0.0045 to 9.1 μM	0.0015 μM	Fruit drink, orange flavored candy, orange flavored jelly powder, cheese snack	[125]
	rGO-g-CN/ZnO/AuNPs/GCE	0.005 to 0.085 μM	0.0013 μM	Candy, fruit juice, red wine	[126]
	MIPs/f-MWCNTs/GCE	0.05 to 100 µM	5.0 nM	Candy, orange-flavored jelly powder, peach juice powder, candy-coated chocolate, beverage	[107]
	rGO/CPE	0.05 to 10.0 μM	0.027 μM	Fanta, Mirinda, cheese snack, cheetos	[127]
	Au/RGO/GCE	0.002 to 109.14 μM	2 nM	Fanta, XiangChengDuo, Mirida	[128]
	GO/MWCNTs/GCE	0.09–8.0 μM	0.025 μM	Orange juice	[114]
	ZnO/Cysteic acid/ GCE	0.1 to 3.0 μM	0.03 μM	Orange juice, fruit juice, peach gelatin	[129]
	rGO/NiBTC/SPCEs	0.05 to 5.0 μM	0.025 μM	Orange sports drink, yellow sports drink	[130]
	NiFe_2_O_4_/IL/rGO/ CPE	0.05 to 30.0 μM; 30.0 to 500.0 μM	0.03 μM	Orange juice powder	[131]
Orange II	NH_2_-fMWCNTs/ZnO NPs/GCE	0.03 to 0.09 μM	0.57 nM	Industrial wastewater, fruit juice, ketchup sauce	[111]
	Cbz-AgNP/GCE	1.56 nM to 12 μM	1.2 nM	Drinking water, tap water, fruit juice	[117]
	Fe_2_O_3_/MWCNTs-COOH/OP/CPE	0.2 to 50.0 μM	0.1 μM	Industrial wastewater	[119]
	ERGO-ATDT-Pt/GCE	10 to 600 nM	0.34 nM	Chili sauce, ketchup samples	[113]
	Graphene–mesoporous TiO_2_/GCE	2.85 nM to 0.48 μM	0.92 nM	Chili powder, chili paste, ketchup	[12]
Orange G	Fe_2_O_3_/MWCNTs-COOH/OP/CPE	0.1 to 20.0 μM	0.05 μM	Industrial wastewater	[119]
Methyl orange	Surfactant-intercalated smectite clay/GCE	0.1 to 1.6 μM	40 nM	Natural water	[118]
	PET/AgNPs/GCE	0.02 to 50 μM	7.6 nM	-	[132]
	Poly(L-serine)/CPE	20.0 nM to 1 μM	26 nM	Tap water	[133]
	PRFMCPS	0.6 to 8.0 μM	0.699 μM	Water sample	[105]
	23Fe-21Cr-18Ni-20Ti-18Mn/CPE	1 to 4 mM	0.080 μM	-	[134]
Basic orange 2	Graphene/AgNPs/MIP	3 nM to 50 μM	1 nM	-	[135]
Acid orange 10	TDA/AuE	1 pM to 1 mM	1 pM	Industrial wastewater	[102]
Basic orange 1	MGO/β-CD@AuNPs/GCE	0.05 to 50 μM	17 nM	Water sample	[108]
	PDDA-MWCNTs/ds-DNA)/MIP/PGE	0.05 to 15.00 μg/mL	0.03 μg/mL	Fish, sauces (chili and ketchup), textile effluents	[136]
	Fe_3_O_4_@SiO_2_/thylene glycol dimethacrylate	2 to 100 μM	0.619 μM	Yellow croaker, yuba (bean curd skin)	[137]
Disperse orange 1	DO1-BSA/anti-DO1/HRP	-	0.87 ng/mL	-	[138]
	GCE/1,12-diaminododecane/1,7-diaminoheptane/anti-DO1 antibodies	5.0 nM to 0.5 μM	7.56 nM	Tap water	[139]
	La_10_Si_6_O_27_:Tb^3+^/CPE	1 to 5 mM	-	-	[140]
	poly(glutamic acid)/GCE	0.25 to 3 μM	15 nM	Textile industry wastewater sample	[141]
Solvent orange 7	GCE	4 to 18 μM	0.9 μM	Commercial fuel ethanol sample	[100]
	PASA/GCE	0.04 to 1 µM; 1 to 12 µM	4 nM	Chili powder, chili sauce, ketchup	[104]
	ZnONPs/CPE	0.01 to1.0 μM and 1.0 to 20.0 μM	1.7 nM	Chili, ketchup sauce	[142]
	MWNTs-IL-Gel/GC	0.005 to 20 μg/mL	0.001 μg/mL	Chili, ketchup sauce	[143]
	AGCE	0.4 to 10 μM	80 nM	Chili powder, chili sauce, strawberry sauce, tomato sauce	[144]
	DTT/SPGE	1 nM to 1.5 μM	2 nM	Ketchup, chili sauce, salsa dip sauce	[145]
	ds-DNA/AuNPs/poly-(o-phenylenediamine)/PGE	1 to 20 nM and 20 to 500 nM	0.3 nM	Chili, ketchup sauce	[146]
	ZnONPs/CPE	0.05 to 20.0 μM	1.87 nM	Chili sauce, ketchup samples	[147]
Natural orange 6	GCE	0.60 to 1.40 μM	6 nM	Leaf, fruit, and bark extracts of the plant Lawsonia inermis	[148]
	Boron-doped diamond electrode	0.1 to 5.0 μM	29 nM	Commercial henna samples	[149]

### 4.4. Biological-Based Electrochemical Biosensors

DNA interaction refers to the binding or association between DNA molecules and other substances, such as small molecules, proteins, or drugs. This interaction can occur through various mechanisms, including intercalation, groove binding, or electrostatic interactions with the DNA backbone. These interactions can be harnessed for electrochemical sensing because they often result in measurable changes to the electrochemical properties of the system. When target molecules bind to DNA, they can alter its structure, conformation, or electron transfer capabilities, which can be detected using electrochemical techniques. In electrochemical DNA sensors, DNA is typically immobilized on an electrode surface. The interaction between the immobilized DNA and target analytes can be measured through changes in electrical signals, such as current, potential, or impedance.

In regard to orange dye detection, Ensafi et al. [136] developed an electrochemical sensor for detecting basic orange 1 based on its interaction with DNA. They used a pencil graphite electrode modified with MWCNT and double-stranded DNA. The electrode was prepared by attaching MWCNTs to the surface using poly(diallyldimethylammonium chloride), followed by the immobilization of dsDNA. When exposed to basic orange 1, the dye molecules interacted with the immobilized DNA, likely through intercalation or groove binding. The interaction between basic orange 1 and DNA was detected through two main electrochemical signals: a decrease in the oxidation peaks of guanine and adenine bases in the DNA, and an increase in the oxidation signal of basic orange 1 itself. These changes in electrochemical signals were proportional to the concentration of basic orange 1, allowing for its quantitative determination. The DNA-based electrochemical sensor demonstrated high sensitivity and selectivity for basic orange 1, with a low LOD and wide linear range. Ferreyra and Rivas [150] investigated the interaction between acridine orange and calf-thymus double stranded DNA (dsDNA) in supramolecular architectures on gold electrodes. The researchers built multilayers of polyethylenimine (PEI) and dsDNA through self-assembly. They found that pH and ionic strength significantly influenced the multilayer formation. The interaction between acridine orange and confined dsDNA was evaluated by measuring the acridine orange oxidation signal. The amount of accumulated acridine orange increased linearly with up to three PEI-dsDNA bilayers, but became more difficult to electrooxidize in higher numbers of bilayers.. The study demonstrated selectivity by comparing responses between dsDNA and single-stranded DNA multilayers. For structures with 2–6 bilayers, the AO oxidation current rapidly saturated at concentrations above 0.5 mM. A similar work was also conducted by Gherghi et al. [151]. Uliana et al. [152] investigated the interactions between DNA and disperse orange 1 using an electrochemical DNA biosensor. They found that disperse orange 1 and its electrolysis products caused significant decreases in the oxidation signals of guanine and adenine bases in DNA. Disperse orange 1 showed a more pronounced effect, reducing the guanine and adenine signals by 48% and 51%, respectively, at a 1 μM concentration. The study also revealed evidence of DNA structural changes and damage, including the appearance of new voltametric peaks and shifts in peak potentials.

**Figure 8:**
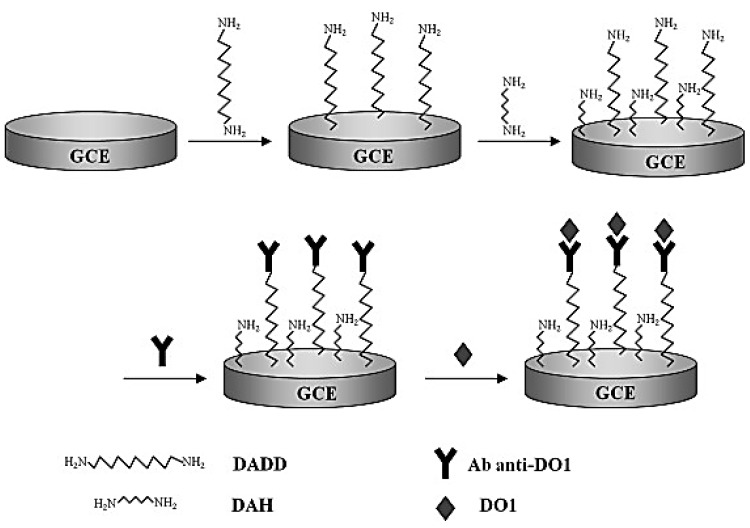
Schematic representation of the preparation procedure and detection principle of the immunosensor [139].

An immunosensor is a specialized analytical device that utilizes antibody–antigen interactions to detect specific target molecules. In a study reported by Uliana and Yamanaka [138], they developed an immunosensor for detecting disperse orange 1. The sensor employed a unique non-conventional competitive assay approach, combining magnetic particle separation and amperometric detection. The construction of this immunosensor involved several key components. Gold electrodes were modified with a conjugate of disperse orange 1 and bovine serum albumin (DO1-BSA). Magnetic particles were functionalized with both anti-DO1 antibodies and horseradish peroxidase (HRP) enzymes. This dual-functionalization allowed for both target capture and signal amplification. The sensing mechanism relied on competition between disperse orange 1 molecules immobilized on the electrode surface and free disperse orange 1 in the sample for binding to the antibodies on the magnetic particles. As the disperse orange 1 concentration in the sample increased, fewer antibody-coated magnetic particles could bind to the electrode surface, resulting in a decreased amperometric signal. They employed a multi-step process for detection. First, they captured disperse orange 1 from the sample using the functionalized magnetic particles. Then, they brought these particles into contact with the modified gold electrodes. The competitive binding occurred, and finally, they measured the amperometric response using a system involving HRP, hydrogen peroxide, and hydroquinone. In terms of performance, this immunosensor demonstrated impressive capabilities. It achieved a low LOD of 0.87 ng/mL for disperse orange 1, indicating high sensitivity. The sensor exhibited a linear response over a range of disperse orange 1 concentrations, allowing for quantitative analysis. The use of magnetic particles for sample cleanup and preconcentration enhanced the sensor’s ability to detect disperse orange 1 in complex mixtures. Additionally, the HRP labeling provided signal amplification, contributing to the high sensitivity. Yang et al. [139] developed a label-free impedimetric immunosensor for detecting the textile dye disperse orange 1. They modified a GCE by grafting 1,12-diaminododecane and 1,7-diaminoheptane, then immobilizing anti-DO1 antibodies (Figure 8). The immunosensor’s construction and performance were characterized using cyclic voltammetry, electrochemical impedance spectroscopy, and capacitance measurements. The sensor exhibited a linear response to disperse orange 1 concentrations ranging from 5.0 nM to 0.5 μM, with a correlation coefficient of 0.9980. The LOD was determined to be 7.56 nM. The immunosensor demonstrated good reproducibility with a relative standard deviation of 2.66% for three measurements. When applied to tap water samples spiked with 1.25 μM disperse orange 1, the sensor achieved recovery rates between 96.78% and 110.94%.

Table 3 provides a comprehensive overview comparing nanomaterial-based and biological-based sensors across these important parameters. In general, nanomaterial-based sensors tend to offer a higher sensitivity and stability, with detection limits often in the nanomolar range and shelf lives of several months. Their selectivity can be moderate to high, depending on the specific nanomaterials and surface modifications used. The cost of nanomaterial-based sensors is typically moderate, with some high-end materials increasing the overall expense. On the other hand, biological-based sensors, such as those utilizing DNA or antibodies, generally exhibit excellent selectivity due to the specific biomolecular interactions involved. However, their stability is often lower compared to nanomaterial-based sensors, with shorter shelf lives and potential sensitivity to environmental conditions. The sensitivity of biological-based sensors can vary widely, from nanomolar to micromolar detection limits, depending on the specific biorecognition element and transduction method used. In terms of cost, biological-based sensors can range from moderate to high, particularly when using specialized antibodies or aptamers.

## 5. Application-Specific Sensor Platforms

The diverse range of electrochemical sensing platforms for synthetic orange dyes offers unique advantages for different applications. This section provides a critical comparison of the sensor platforms best suited for food safety, environmental monitoring, and smart packaging applications.

In food safety, high selectivity and sensitivity are paramount due to the complex matrices of food samples and the need to detect low concentrations of potentially harmful dyes. MIP-based sensors have shown exceptional promise in this area. For instance, the RMIEC developed by Wu et al. [106] for food yellow 3 detection achieved a remarkably low detection limit of 1.60 nM with high selectivity. This sensor demonstrated an excellent performance in real food samples like Mirinda orange and Fanta orange, making it highly suitable for food safety applications. Nanomaterial-based sensors also play a crucial role in food safety monitoring. The graphene–mesoporous TiO_2_ nanocomposite-modified GCE developed by Gan et al. [120] for Orange II detection achieved a low detection limit of 0.92 nM and was successfully applied to food samples such as chili powder and ketchup. The high surface area and catalytic properties of nanomaterials contribute to enhanced sensitivity, which is essential for detecting trace amounts of dyes in food products. For rapid on-site testing in food processing facilities, screen-printed electrodes offer practical advantages. Wu and Lee [130] developed a screen-printed carbon electrode modified with reduced graphene oxide and NiBTC frameworks for the simultaneous determination of food yellow 3 and tartrazine in drinks. This type of sensor combines the benefits of nanomaterials with the portability and ease of use required for on-site testing.

Environmental monitoring often requires sensors capable of detecting a wide range of concentrations in complex water matrices. Nanomaterial-based sensors are particularly well-suited for this application due to their broad linear ranges and high sensitivity. For example, the NH2-fMWCNTs/ZnO NPs/GCE sensor developed by Irfan et al. [111] for Orange II detection demonstrated a remarkably low detection limit of 0.57 nM and was successfully applied to industrial wastewater samples. Composite nanomaterial sensors have shown promise in the simultaneous detection of multiple dyes in environmental samples. Liu et al. [119] developed a Fe_2_O_3_/MWCNTs-COOH/OP/CPE sensor capable of simultaneously detecting Orange G and Orange II in industrial wastewater, with detection limits of 0.05 μM and 0.1 μM, respectively. This multi-analyte detection capability is particularly valuable for comprehensive environmental monitoring. For field testing of water quality, sensors based on unmodified electrodes may offer advantages in terms of robustness and simplicity. The glassy carbon electrode used by Sierra-Rosales et al. [97] for studying the electrooxidation of food yellow 3 provides insights into the fundamental behavior of dyes in aqueous environments, which is crucial for developing effective monitoring strategies.

Smart packaging requires sensors that are not only sensitive and selective but also cost-effective and easily integrable into packaging materials. In this context, screen-printed electrodes and flexible sensor designs show great potential. While not specifically designed for orange dyes, the battery-free and wireless epidermal electrochemical system with an all-printed stretchable electrode array developed by Xu et al. [73] demonstrates the potential for integrating electrochemical sensors into flexible packaging materials. For smart packaging applications targeting orange dyes, adapting approaches like the poly(L-serine)-modified carbon paste electrode developed by Nikhil et al. [133] for methyl orange detection could be promising. This type of sensor combines good sensitivity with potentially low-cost materials suitable for large-scale production in packaging applications. Colorimetric sensors that can be visually interpreted may also play a role in smart packaging. While not an electrochemical method, the principles behind the plasmonic nanoparticle-based SERS platform developed by Wu et al. [9] for detecting Orange II could inspire the development of dual-mode sensors combining electrochemical and optical detection for smart packaging applications.

The choice of sensor platform depends heavily on the specific requirements of each application. MIP-based and nanomaterial-enhanced sensors are particularly well-suited for food safety applications due to their high selectivity and sensitivity. Environmental monitoring benefits from the wide linear ranges and multi-analyte detection capabilities of composite nanomaterial sensors. Smart packaging applications favor cost-effective and easily integrable designs, such as screen-printed and flexible electrodes. As research progresses, we anticipate the development of more versatile sensors that can address the unique challenges of each application area while maintaining a high performance across various sample matrices.

The commercial potential for electrochemical sensors targeting synthetic orange dyes in food safety applications is significant, driven by increasing regulatory scrutiny and consumer demand for safer food products. However, the path to commercialization requires careful consideration of several key factors. Regulatory approval is a critical step in bringing these sensors to market. In the United States, for example, sensors intended for food safety applications must comply with FDA regulations for food contact materials. This process typically involves demonstrating that the sensor components do not migrate into food products and that the detection limits meet or exceed the current regulatory standards for synthetic dyes. The sensitivity of our MIP-based sensors, such as the RMIEC developed by Wu et al. [106] with a detection limit of 1.60 nM for food yellow 3, positions them well to meet these requirements. Cost-effectiveness is another crucial consideration. While nanomaterial-based sensors offer a superior performance, their production costs must be competitive with existing detection methods to gain market traction. One promising approach is the use of screen-printing techniques for large-scale sensor production, which could significantly reduce manufacturing costs. For instance, the screen-printed carbon electrode modified with reduced graphene oxide and NiBTC frameworks developed by Wu and Lee [130] demonstrates the potential for cost-effective, mass-producible sensors. Scalability and integration into existing food processing systems are essential for widespread adoption. Sensors based on unmodified electrodes, such as the glassy carbon electrode used by Sierra-Rosales et al. [97], offer advantages in terms of robustness and simplicity, making them more readily adaptable to industrial environments. For more advanced sensors, we envision developing modular units that can be easily integrated into existing food production lines for continuous, inline monitoring.

## 6. Challenges and Future Perspectives

### 6.1. Matrix Effects in Complex Samples

While significant progress has been made in developing electrochemical sensors for synthetic orange dyes, several challenges persist that limit their widespread application in complex real-world samples. Two key issues that deserve particular attention are matrix effects in complex samples and long-term stability concerns. Matrix effects pose a significant challenge for the accurate detection of synthetic orange dyes in food and environmental samples. These effects arise from the presence of numerous interfering compounds that can interact with the electrode surface or the target analyte, leading to altered electrochemical responses. In food samples, various components can interfere with dye detection:

- Proteins: In milk-based products, caseins can adsorb onto electrode surfaces, forming a barrier that impedes electron transfer.
- Lipids: In fatty foods, lipid molecules can form an insulating layer on the electrode surface.
- Carbohydrates: Sugars present in fruit juices and soft drinks can interfere with dye adsorption on electrode surfaces.
- Polyphenols: Common in beverages like tea and wine, polyphenols can compete with dye molecules for adsorption sites.
- Preservatives: Sulfites, commonly used in processed foods, can directly interact with azo dyes.

Environmental samples present a different set of matrix effects:

- Humic substances: In water samples, humic and fulvic acids can form complexes with dye molecules.
- Inorganic ions: High salt concentrations in wastewater can lead to increased background currents.
- Surfactants: Commonly present in industrial effluents, surfactants can alter the electrode–solution interface.
- Heavy metals: Industrial wastewater often contains heavy metals that can interfere with dye detection.

Unmodified electrodes tend to suffer from rapid fouling in complex matrices, leading to significant signal degradation after just a few measurements in food samples. Their long-term stability in clean solutions is generally good, but sensitivity often declines due to surface oxidation. Chemically modified electrodes, particularly those using molecularly imprinted polymers, show improved resistance to matrix effects but may experience a gradual loss of recognition sites over repeated use. Their stability can vary widely depending on the specific modifying layer, with some polymer coatings maintaining performance for several months while others degrade within weeks. Nanomaterial-based sensors often exhibit an excellent initial performance in complex samples due to their high surface area and catalytic properties. However, they can be particularly susceptible to long-term stability issues. For instance, carbon nanotube-based sensors may show a 20–30% decrease in sensitivity after a month of storage due to aggregation and surface oxidation. Gold nanoparticle-modified electrodes can maintain their performance for several months in ideal conditions but may rapidly degrade when exposed to samples containing high levels of chloride ions or sulfur-containing compounds. Biological-based sensors, such as those using DNA or enzymes, typically offer high selectivity but are the most vulnerable to environmental factors. Enzyme-based sensors may lose 50% or more of their initial activity within a week when stored at room temperature. DNA-based sensors are generally more stable but can still experience significant degradation in performance when exposed to nucleases present in some food and environmental samples.

### 6.2. Future Research Directions

Future research in the electrochemical sensing of synthetic orange dyes is likely to focus on addressing the current limitations while exploring new avenues for improved performance. One emerging trend is the development of multi-modal sensing platforms that combine electrochemical detection with other analytical techniques, such as spectroscopic methods. These hybrid approaches could provide complementary information, enhancing both the selectivity and reliability of dye detection.

Advanced nanomaterials and nanocomposites are expected to play a crucial role in future sensor designs. The exploration of novel 2D materials beyond graphene, such as transition metal dichalcogenides or MXenes, may lead to sensors with improved stability and sensitivity. Additionally, the integration of stimuli-responsive materials could enable the development of smart sensors capable of adapting to different sample matrices or environmental conditions.

Sensor miniaturization represents a significant frontier in the development of electrochemical sensors for synthetic orange dyes, particularly for on-site testing applications. The ability to reduce sensor size while maintaining or even improving performance characteristics is critical for expanding the utility of these devices beyond laboratory settings. Miniaturized sensors offer numerous advantages, including reduced sample volume requirements, faster response times, and increased portability. These features are especially valuable in food safety and environmental monitoring contexts, where rapid, in-field analysis can significantly impact decision-making processes. Recent advancements in microfabrication techniques and nanomaterials have opened new avenues for sensor miniaturization. For instance, the integration of microfluidic channels with electrochemical detection systems has shown promise in creating lab-on-a-chip devices capable of detecting analytes at very low concentrations [153]. These miniaturized systems not only reduce the overall footprint of the sensing platform but also enhance its sensitivity through precise control of the sample flow and reaction conditions.

Artificial intelligence (AI) and machine learning (ML) algorithms are increasingly being integrated into electrochemical sensing systems for orange dyes, offering significant improvements in sensitivity, selectivity, and data interpretation. Recent studies have demonstrated the potential of these computational approaches in enhancing sensor performance. For instance, Zhao et al. [154] developed a convolutional neural network (CNN) model to process SERS data from food yellow 3 detection. The CNN was able to extract subtle features from the SERS spectra, allowing for accurate quantification of the dye at concentrations as low as 1 nM. This approach was particularly effective in distinguishing Sunset Yellow signals from interfering species in complex food matrices. Support vector machines (SVMs) have also shown promise in improving the selectivity of multi-analyte sensors. Amsaraj and Mutturi [155] utilized an SVM classifier to differentiate between various orange dyes based on FTIR spectra. Furthermore, AI algorithms can be employed to optimize sensor design and fabrication. Genetic algorithms and reinforcement learning techniques have been used to fine-tune spectrophotometric determination [156]. The processing of data signals in these fields has great potential to be used for the electrochemical detection of orange dyes.

The commercial potential for electrochemical sensors targeting synthetic orange dyes is significant, particularly in the food industry and environmental monitoring sectors. As regulatory scrutiny of synthetic dyes intensifies globally, there is a growing market for rapid, cost-effective screening tools that can be used by food manufacturers, regulatory agencies, and environmental monitoring organizations.

## 7. Conclusions

The rapid evolution of electrochemical sensing strategies for synthetic orange dyes has opened new avenues for addressing critical challenges in food safety, environmental monitoring, and industrial quality control. While significant progress has been made in developing highly sensitive and selective sensors, several pressing issues require urgent attention from the research community and industry stakeholders. One of the most critical challenges is the development of robust sensors capable of reliable performance in complex real-world matrices. Future research should focus on innovative surface modification techniques and advanced nanomaterials that can effectively mitigate matrix effects without compromising sensitivity or selectivity. There is also a pressing need for multi-modal sensing platforms that can provide orthogonal confirmation of results, thereby enhancing the reliability of dye detection in diverse sample types.

The integration of artificial intelligence and machine-learning algorithms into electrochemical sensing systems represents a promising frontier. These computational approaches have the potential to dramatically improve signal processing, pattern recognition, and multi-analyte discrimination. However, significant work is needed to develop standardized datasets and validated algorithms that can be readily adopted by the broader scientific community and industry. Miniaturization and the development of portable, field-deployable sensors remain key industry-driven challenges. There is an urgent need for cost-effective, user-friendly devices that can provide rapid, on-site analysis of synthetic orange dyes in various settings, from food processing facilities to environmental monitoring stations. This will require interdisciplinary collaboration between electrochemists, materials scientists, and engineers to overcome current limitations in sensor design and fabrication. The commercial potential of electrochemical sensors for synthetic orange dyes is substantial, but realizing this potential will require addressing several hurdles. Regulatory agencies, industry associations, and academic researchers must work together to establish standardized testing protocols and performance benchmarks for these sensors. Additionally, efforts should be made to develop scalable manufacturing processes that can translate laboratory prototypes into commercially viable products without sacrificing performance or reliability.

## Figures and Tables

**Table 1:** List of common orange dyes with their structures.

C.I. Generic Name	Common Name	C.I. Number	Class	Structure
Acid orange 3	Acid orange 3	10,385	Nitro	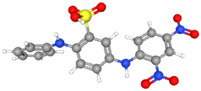
Acid orange 19	Acid orange 19	14,690	Azo	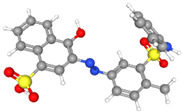
Acid orange 20	Orange I	14,600	Azo	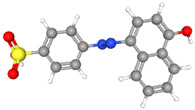
Basic orange 1	Chrysoidine R	11,320	Azo	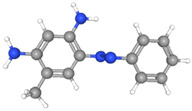
Basic orange 2	Chrysoidine Y	11,270	Azo	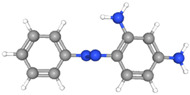
Basic Orange 14	Acridine orange	46,005	Acridine	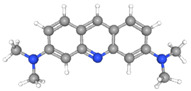
Mordant orange 1	Alizarin yellow R	14,030	Azo	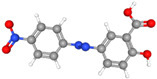
Food orange 6	Apocarotenal	40,820	Carotenoid	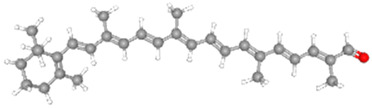
Food orange 7	Food orange 7	40,825	Carotenoid	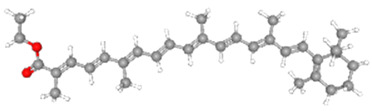
Food orange 8	Canthaxanthin	40,850	Carotenoid	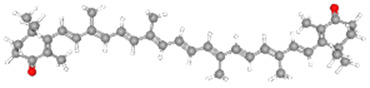
Vat orange 1	Vat orange 1	59,105	Anthraquinone	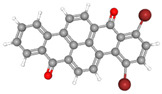
Vat orange 3	Dibromoanthanthrone	59,300	Anthanthrone	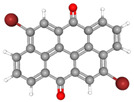
Natural orange 6	Lawsone	75,480	Natural	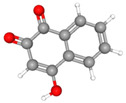
Disperse orange 1	Disperse orange 1	11,080	Azo	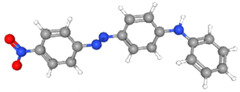
Disperse orange 3	Disperse orange 3	11,005	Azo	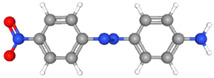
Disperse orange 11	Disperse orange 11	60,700	Anthraquinone	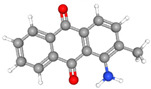
Disperse orange 13	Disperse orange 13	26,080	Diazo	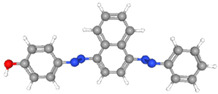
Acid orange 5	Tropaeolin OO	13,080	Azo	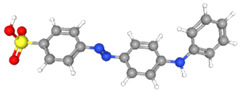
Acid orange 6	Tropaeolin O	14,270	Azo	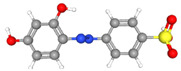
Acid orange 7	Tropaeolin OOO2	15,510	Azo	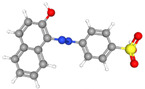
Acid orange 10	Orange G	16,230	Azo	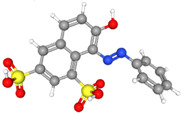
Acid orange 52	Methyl orange	13,025	Azo	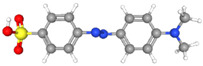
Solvent orange 2	Oil orange SS	12,100	Azo	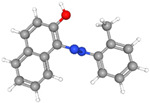
Solvent orange 7	Sudan II	12,140	Azo	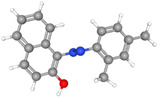
Solvent orange 86	Quinizarin	58,050	Anthraquinone	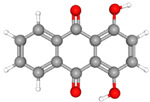
Food yellow 3	Sunset yellow FCF	15,985	Azo	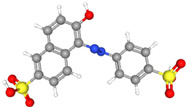

**Table 3:** Comparison of nanomaterial-based sensors and biological-based sensors for synthetic orange dye detection.

Sensor Type	Dye	Detection Limit	Linear Range	Ref.
Nanomaterial-based: NH_2_-fMWCNTs/ZnO NPs/GCE	Orange II	0.57 nM	0.03 to 0.09 μM	[111]
Nanomaterial-based: Cbz-AgNP/GCE	Orange II	1.2 nM	1.56 nM to 12 μM	[117]
Nanomaterial-based: ERGO-ATDT-Pt/GCE	Orange II	0.34 nM	10 to 600 nM	[113]
Nanomaterial-based: Graphene–mesoporous TiO_2_/GCE	Orange II	0.92 nM	2.85 nM to 0.48 μM	[12]
Biological-based: DO1-BSA/anti-DO1/HRP	Disperse orange 1	0.87 ng/mL	-	[138]
Biological-based: GCE/1,12-diaminododecane/1,7-diaminoheptane/anti-DO1 antibodies	Disperse orange 1	7.56 nM	5.0 nM to 0.5 μM	[139]

## Data Availability

Data are contained within the article.
